# Distinguishing Between Long-Transient and Asymptotic States in a Biological Aggregation Model

**DOI:** 10.1007/s11538-023-01254-0

**Published:** 2024-02-11

**Authors:** Jonathan R. Potts, Kevin J. Painter

**Affiliations:** 1 School of Mathematics and Statistics, University of Sheffield, Hounsfield Road, Sheffield, S3 7RH UK; 2 Dipartimento Interateneo di Scienze, Progetto e Politiche del Territorio (DIST), Politecnico di Torino, Turin, Italy

**Keywords:** Aggregation–diffusion equation, Asymptotics, Biological aggregation, Long transients, Metastability, Nonlocal advection

## Abstract

Aggregations are emergent features common to many biological systems. Mathematical models to understand their emergence are consequently widespread, with the aggregation–diffusion equation being a prime example. Here we study the aggregation–diffusion equation with linear diffusion in one spatial dimension. This equation is known to support solutions that involve both single and multiple aggregations. However, numerical evidence suggests that the latter, which we term ‘multi-peaked solutions’ may often be long-transient solutions rather than asymptotic steady states. We develop a novel technique for distinguishing between long transients and asymptotic steady states via an energy minimisation approach. The technique involves first approximating our study equation using a limiting process and a moment closure procedure. We then analyse local minimum energy states of this approximate system, hypothesising that these will correspond to asymptotic patterns in the aggregation–diffusion equation. Finally, we verify our hypotheses through numerical investigation, showing that our approximate analytic technique gives good predictions as to whether a state is asymptotic or transient. Overall, we find that almost all twin-peaked, and by extension multi-peaked, solutions are transient, except for some very special cases. We demonstrate numerically that these transients can be arbitrarily long-lived, depending on the parameters of the system.

## Introduction

Aggregation phenomena are widespread in biology, from cell aggregations (Budrene and Berg 1995) to the swarming (Roussi 2020), schooling (Makris et al. 2009), flocking (Clark and Mangel 1984), and herding (Bond et al. 2019) of animals. When modelled from a continuum perspective (as opposed to via interacting particles), the principal tools take the form of partial differential equations with non-local advection, sometimes combined with a diffusive term (Topaz et al. 2006). Indeed, such equations are often called aggregation equations (Laurent 2007), highlighting their importance in modelling aggregations, or aggregation–diffusion equations (Carrillo et al. 2019) if there is a diffusion term.

As well as modelling aggregated groups of organisms, such equations have also been used to model aggregation-like phenomena elsewhere, such as animal home ranges and territories (Briscoe et al. 2002; Potts and Lewis 2016) and consensus convergence in opinion dynamics (Garnier et al. 2017). This very broad range of applications, together with the mathematical complexity in dealing with nonlinear nonlocal partial differential equations (PDEs), has led to a great amount of interest from applied mathematicians in understanding the properties of these PDEs (Painter et al. 2023).

Of particular interest from a biological perspective are the pattern formation properties of aggregation–diffusion equations, since these can reveal the necessary processes required for observed patterns to emerge. Many analytic techniques for analysing pattern formation, such as linear stability analysis and weakly nonlinear analysis, focus on the onset of patterns from small perturbations of a non-patterned (i.e. spatially homogeneous) state. However, patterns observed in actual biological systems will often be far from the non-patterned state, and not necessarily emerge from small perturbations of spatially homogeneous configurations (Krause et al. 2020; Veerman et al. 2021a).

Sometimes observed patterns will be asymptotic steady states or other types of attractors. But frequently biological systems will be observed in transient states (Hastings et al. 2018; Morozov et al. 2020). These transient states may persist for a very long time, sometimes so long that they are hard to distinguish from asymptotic states. As in previous studies (e.g. Morozov et al. 2020), we refer to such solutions as ‘long transient’ solutions (noting that we make no pretence of rigour in the definition of ‘long’, but simply using the word heuristically to highlight the sort of solutions that are likely to be mistaken for asymptotic steady states without unusually thorough analysis). As well as long transients being difficult to decipher from observations of biological systems, they can also be tricky to determine from numerical solutions of a PDE model. Therefore analytic techniques are required to guide those engaging in numerical analysis of PDEs [including continuation methods (Uecker 2022)] as to whether the solution they are observing is likely to be a long transient or an asymptotic state.

Our aim here is to provide such analytic techniques for a class of 1D aggregation-diffusion equations of the following form

1 $$\begin{aligned} \frac{\partial u}{\partial t}=D\frac{\partial ^2 u}{\partial x^2}-\gamma \frac{\partial }{\partial x}\left[ u\frac{\partial }{\partial x}(K*u)\right] , \end{aligned}$$

where *K* is a non-negative averaging kernel, symmetric about 0, with $\Vert K\Vert _{L^1}=1$, and

2 $$\begin{aligned} K*u(x)=\int _{\Omega }K(z)u(x+z{,t})\textrm{d}z \end{aligned}$$

is a convolution, where $\Omega$ is the spatial domain of definition. Here, *D* and $\gamma$ are constants, and $\Omega$ is the circle given by interval $[-L,L]$ with periodic boundary conditions imposed. This choice of boundary conditions is made purely for analytic simplicity, as it is a non-trivial problem to define other types of boundary condition for spatially non-local advection (Hillen and Buttenschön 2020).

Our approach is not exact, in the sense that we approximate our study PDE through the limit as $D/\gamma \rightarrow 0$, and via a moment closure assumption. However, this approximation allows us to analyse the associated energy functional, finding explicit mathematical expressions for local energy minima. Our conjecture is that local energy minima of the approximate system are qualitatively similar to the asymptotic patterns observed in the aggregation-diffusion equation we are studying, but any states that do not represent local energy minima of the approximate system are transient states. We then test this numerically in some specific cases.

Of particular interest is the question of whether multi-peaked solutions are asymptotic steady states or long transients. Various numerical studies of Eq. (1), and similar equations, report multi-peaked solutions (Armstrong et al. 2006; Buttenschön and Hillen 2020; Carrillo et al. 2019; Daneri et al. 2022). However, merging and decaying of peaks have also been observed. Furthermore, analytic investigations into chemotaxis equations, which have some similarities with aggregation equations, have demonstrated that multi-peaked solutions can often be long transients (Potapov and Hillen 2005).

Our work demonstrates that, except for the very specific case where peaks are of identical heights and evenly-spaced, any two-peaked solutions will eventually evolve into a solution with at most one peak, as the smaller peak decays to zero. The time it takes for the smaller peak to decay grows rapidly with the start height of the smaller peak, eventually tending to infinity as the difference in start heights between the two peaks tends to zero. We show that a key parameter governing the speed of this decay is the diffusion constant *D*, with higher diffusion constants leading to faster decays. We conjecture that, as $D \rightarrow 0$, the time to decay tends to infinity, meaning that two-peaked solutions become stable.

Finally, we investigate the effect of incorporating logistic growth of the population into our model, leading to a Fisher–KPP model with non-local advection (e.g. Hamel and Henderson 2020). The motivation for this is that, in situations where transient solutions exists for a long time, it is no longer biologically reasonable to assume that we are working in situations where births and deaths are negligible. We show that, for a given set of parameters and initial condition, there is a critical net reproduction rate, below which the smaller peak will decay and above which it will persist.

This paper is organised as follows. In Sect. 2, we detail our methodological approach. Section 3 deals with minimum energy configurations of single-peaked solutions. Section 4 examines twin-peaked solution, their merging (Sect. 4.1) and decaying (Sect. 4.2) dynamics, and the effect of including population growth (Sect. 4.3). Section 5 gives some discussion and concluding remarks.

## Methodological Approach

**Fig. 1:**
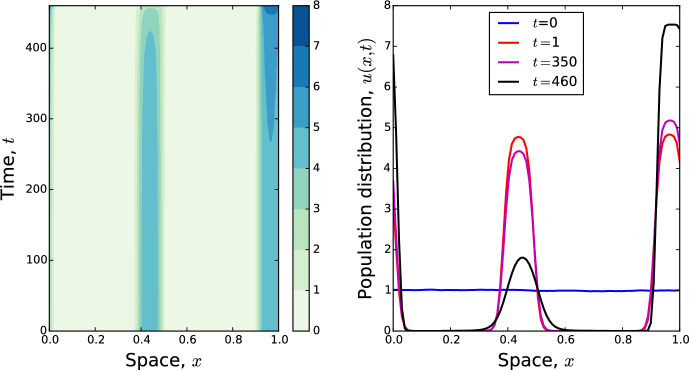
Numerical solutions of Eq. (1) starting with initial conditions that are a small random fluctuation of the constant steady state (noise generated uniformly at random from $[-0.005,0.005]$ at each point in space). By $t=1$ clear aggregations have formed that might seem stable were the solution only run to around time $t=100$. However, if we run the solution further in time, we see that the middle peak is gradually decaying, and this decay is speeding up over time, so that by $t=460$ the peak in the middle is much smaller than the other peak. Here, $D=1$, $\gamma =10$, and *K* is a top-hat kernel (Eq. 19) with $\delta =0.1$

Our study is motivated by an observation. Often, when simulating Eq. (1), multiple aggregations may form and persist for a very long time. This can give the appearance of multi-peaked asymptotic stable states. For example, Fig. 1 shows a numerical solution where two peaks have formed by time $t=1$. These appear stable on timescales up to two orders of magnitude longer than the time they took to form: even by time $t=100$, the solution has not changed very much (Fig. 1a). However, if we keep running the simulation, we see one of the peaks decay and the other slowly swallow up the former’s mass. The question then arises whether multi-peaked solutions to Eq. (1) are ever actually stable, or are they always just long transients?

To answer this question, our approach will start not by analysing Eq. (1) directly, but rather taking two approximations, simplifying the system and thus enabling us to perform exact calculations. From this starting point, we will then drop these approximations and examine Eq. (1) numerically. This will enable us to ascertain the extent to which numerical solutions of Eq. (1) correspond to our analytic insights of the approximate system.

One approximation we make is to consider the limit as $D/\gamma \rightarrow 0$. Further, we assume that *K* has finite second moment and is sufficiently narrow to make the following moment closure approximation

3 $$\begin{aligned} K*u(x{,t})&\approx u+\frac{\sigma ^2}{2}\frac{\partial ^2 u}{\partial x^2} \end{aligned}$$

where

4 $$\begin{aligned} \sigma ^2=\int _{-L}^L x^2K(x)\textrm{d}x \end{aligned}$$

is the second moment of *K*. This leads to the following approximate version of Eq. (1)

5 $$\begin{aligned} \frac{\partial u}{\partial t}=-\gamma \frac{\partial }{\partial x}\left[ u\left( \frac{\partial u}{\partial x}+\frac{\sigma ^2}{2}\frac{\partial ^3 u}{\partial x^3}\right) \right] . \end{aligned}$$

This is a 1D version of an equation recently proposed by Falcó et al. (2023) for modelling biological aggregations. Note that Eqs. (1) and (5) both preserve mass when solved with periodic boundary conditions (i.e. $u({L,t})=u({-L,t})$ and $\frac{\partial u}{\partial x}({L,t})=\frac{\partial u}{\partial x}({-L,t})$), so that if we define

6 $$\begin{aligned} p:=\int _{-L}^{L}u(x,0)\textrm{d}x \end{aligned}$$

then

7 $$\begin{aligned} \int _{-L}^{L}u(x,t)\textrm{d}x = p, \end{aligned}$$

for all $$t>0$$.

Our tactic will be to search for minimum energy solutions to Eq. (5) using the following Cahn–Hilliard type energy functional

8 $$\begin{aligned} E[u]=-\int _{-L}^L u\left( u+\frac{\sigma ^2}{2}\frac{\partial ^2 u}{\partial x^2}\right) \textrm{d}x. \end{aligned}$$

This type of energy is well-known to be non-increasing in time and unchanging when the system is at steady state (e.g. Novick-Cohen 1988). However, for the sake of completeness, we include the relevant calculation as follows

9 $$\begin{aligned} \frac{\partial E}{\partial t}&=-\int _{-L}^L \left[ \frac{\partial u}{\partial t}\left( u+\frac{\sigma ^2}{2}\frac{\partial ^2 u}{\partial x^2}\right) +u\left( \frac{\partial u}{\partial t}+\frac{\sigma ^2}{2}\frac{\partial ^2 }{\partial x^2}\frac{\partial u}{\partial t}\right) \right] \textrm{d}x \nonumber \\&=-\int _{-L}^L 2\frac{\partial u}{\partial t}\left( u+\frac{\sigma ^2}{2}\frac{\partial ^2 u}{\partial x^2}\right) \textrm{d}x \nonumber \\&=2\gamma \int _{-L}^L \frac{\partial }{\partial x}\left[ u\frac{\partial }{\partial x}\left( u+\frac{\sigma ^2}{2}\frac{\partial ^2 u}{\partial x^2}\right) \right] \left( u+\frac{\sigma ^2}{2}\frac{\partial ^2 u}{\partial x^2}\right) \textrm{d}x \nonumber \\&=-2\gamma \int _{-L}^L u\frac{\partial }{\partial x}\left( u+\frac{\sigma ^2}{2}\frac{\partial ^2 u}{\partial x^2}\right) \frac{\partial }{\partial x}\left( u+\frac{\sigma ^2}{2}\frac{\partial ^2 u}{\partial x^2}\right) \textrm{d}x \nonumber \\&=-2\gamma \int _{-L}^L u\left[ \frac{\partial }{\partial x}\left( u+\frac{\sigma ^2}{2}\frac{\partial ^2 u}{\partial x^2}\right) \right] ^2 \textrm{d}x. \end{aligned}$$

Here, the second and fourth equalities use integration by parts, together with the periodic boundary conditions. We are interested in non-negative solutions to Eq. (5), as these are biologically relevant. Such solutions are known to exist in general (e.g. Novick-Cohen and Segel 1984; Chen et al. 2019), and in particular were always observed in our numerical experiments with non-negative initial data. In this case the final expression in Eq. (9) is non-positive, so that *E*[*u*] is non-increasing and zero when the system is at steady state.

Equation (9) shows that critical points, $u_*(x)$, of the energy functional occur when

10 $$\begin{aligned} \int _{-L}^L u_*\left[ \frac{\partial }{\partial x}\left( u_*+\frac{\sigma ^2}{2}\frac{\partial ^2 u_*}{\partial x^2}\right) \right] ^2 \textrm{d}x = 0, \end{aligned}$$

which means that, on any connected subset of $[-L,L]$, either $u_*(x)=0$ or

11 $$\begin{aligned}&u_*+\frac{\sigma ^2}{2}\frac{\partial ^2 u_*}{\partial x^2} = C \nonumber \\&\quad \implies u_*(x)=C+A\sin \left( \frac{x\sqrt{2}}{\sigma }\right) +B\cos \left( \frac{x\sqrt{2}}{\sigma }\right) \end{aligned}$$

for constants *A*, *B*, and *C*. These constants must be picked so that the initial and boundary conditions on *u*(*x*, *t*) are met, but other than that they are arbitrary. Part of the aim moving forwards is to determine which constants minimise the energy in different specific situations.

To make our search for minimum energy solutions tractable, our investigations are informed by observations from numerical solutions. These numerics suggest that Eq. (1) tends towards a solution containing one or many aggregations, interspersed by constant sections close or near to zero (e.g. Fig. 1). We want to construct differentiable solutions that have this type of qualitative appearance, yet also correspond to critical points of *E*[*u*]. These can be constructed piecewise from Eq. (11). For example, as long as $\pi \sigma <\sqrt{2}L$, a single-peaked solution can be given as follows

12 $$\begin{aligned} u_*(x)={\left\{ \begin{array}{ll} \epsilon +c_\epsilon \left[ 1+\cos \left( \frac{x\sqrt{2}}{\sigma }\right) \right] , &{}\text{ if } x \in \left( -\frac{\pi \sigma }{\sqrt{2}},\frac{\pi \sigma }{\sqrt{2}}\right) \\ \epsilon , &{} \text{ otherwise, } \end{array}\right. } \end{aligned}$$

where $\epsilon \in \left[ 0,\frac{p}{2L}\right]$ and $c_\epsilon$ are constants. One can also construct multi-peaked solutions in a similar way (which we will do later in the case of two peaks). Notice that such solutions are continuously differentiable, i.e. $u_* \in C^1([-L,L])$, but not necessarily twice differentiable, so need to be understood in a weak sense (Evans 2022).

By Eq. (7), a direct calculation gives

13 $$\begin{aligned} c_\epsilon =\frac{p-2\epsilon L}{\sqrt{2}\pi \sigma } \end{aligned}$$

so that the only free parameter in Eq. (12) is $\epsilon$. Since the energy, *E*[*u*], is non-increasing over time, the question arises as to which value of $\epsilon$ minimises *E*[*u*] across the set of all functions of the form in Eq. (12). Our approach is to derive such minima, both in the example from Eq. (12) and in various multi-peaked examples, conjecturing that such minima ought to approximate asymptotic solutions to the original problem in Eq. (1). We then test these conjectures by investigating Eq. (1) numerically.

## Single Peak

Combining Eqs. (8) and (12) gives

14 $$\begin{aligned} E[u_*]=-\int _{-L}^L u_*\left( u_*+\frac{\sigma ^2}{2}\frac{\textrm{d}^2 u_*}{\textrm{d} x^2}\right) \textrm{d}x. \end{aligned}$$

Now, for $-\pi \sigma /\sqrt{2}<x<\pi \sigma /\sqrt{2}$, we have that

15 $$\begin{aligned} u_*(x)=\epsilon +c_\epsilon \left[ 1+\cos \left( \frac{x\sqrt{2}}{\sigma }\right) \right] \end{aligned}$$

which is a solution to

16 $$\begin{aligned} u_*+\frac{\sigma ^2}{2}\frac{\textrm{d}^2 u_*}{\textrm{d} x^2} = \epsilon +c_\epsilon . \end{aligned}$$

Hence

17 $$\begin{aligned} E[u_*]&=-\int _{-\frac{\pi \sigma }{\sqrt{2}}}^{\frac{\pi \sigma }{\sqrt{2}}}\left[ \epsilon +c_\epsilon \left( 1+\cos \left( \frac{\sqrt{2}x}{\sigma }\right) \right) \right] (\epsilon +c_\epsilon )\textrm{d}x-2\int _{\frac{\pi \sigma }{\sqrt{2}}}^L\epsilon ^2\textrm{d}x&\nonumber \\&\quad =-\pi \sigma \sqrt{2}(c_\epsilon ^2+2\epsilon c_\epsilon )-2L\epsilon ^2. \end{aligned}$$

Using Eq. (13) and rearranging gives

18 $$\begin{aligned} E[u_*]=\frac{2L}{\pi \sigma }(\pi \sigma -\sqrt{2}L)\epsilon ^2 + \frac{2p}{\pi \sigma }(\sqrt{2}L-\pi \sigma )\epsilon - \frac{p^2}{\sqrt{2}\pi \sigma }. \end{aligned}$$

Since $\pi \sigma <\sqrt{2}L$ (see above Eq. 12), this is a negative quadratic in $\epsilon$. Furthermore, the maximum is where $\epsilon =\frac{p}{2L}$. Now, $\epsilon \in \left[ 0,\frac{p}{2L}\right]$, so $E[u_*]$ is an increasing function of $\epsilon$ on the interval $\left[ 0,\frac{p}{2L}\right]$. Hence the minimum energy is where $\epsilon =0$. [Note that the same minimum energy configuration for Eq. (5) was found by Falcó et al. (2023, Section 2.3.1). However, there the authors find the energy minimum over a different set of functions from those described by Eq. (12) here. Specifically, they search through compactly supported single-peaked solutions (so $\epsilon =0$) but allow for non-differentiable (but still continuous) solutions. Then the support of $u_*(x)$ is $(-r,r)$ for $0<r\le \pi \sigma /2$ and their search is for the value of *r* that minimises the energy, which they show is $r=\pi \sigma /2$ (in our notation).]

Our analysis suggests that if a numerical solution to either Eq. (1) or (5) results in a single peak at long times, we might expect that peak to be of a similar form to Eq. (12) with $\epsilon =0$. We test this conjecture by solving Eq. (1) numerically with initial conditions given by Eq. (12) for various different values of $\epsilon \in \left[ 0,\frac{p}{2L}\right]$, fixing $p=L=1$. For these simulations, we set $D=1$, $\gamma =10$, and

19 $$\begin{aligned} {K}(x)={\left\{ \begin{array}{ll}\frac{1}{2\delta } &{} \hbox {for}\,-\delta<x<\delta \\ 0 &{} \text{ otherwise, } \end{array}\right. } \end{aligned}$$

so that $$\sigma =\delta /\sqrt{3}$$.

**Fig. 2:**
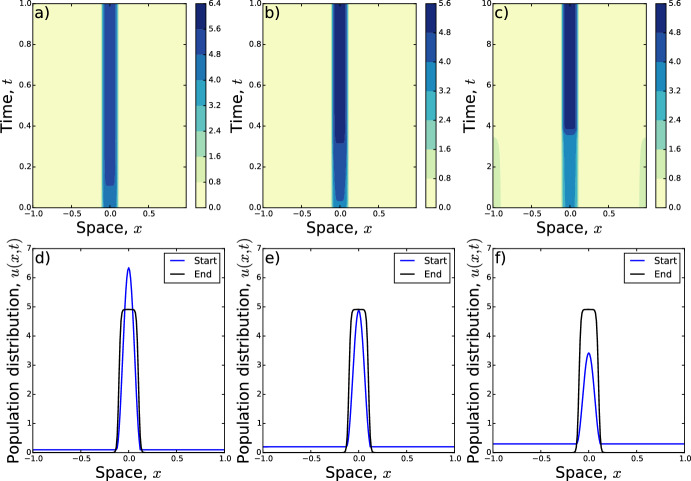
When the initial condition is a single peak surrounded by an area of constant density $\epsilon$, that area becomes sucked-up into the peak. The respective $\epsilon$-values are **a**, **d** $\epsilon =0.1$; **b**, **e** $\epsilon =0.2$; **c**, **f** $\epsilon =0.3$. In **c**, a second peak emerges at $x=\pm 1$ but decays by around $t \approx 4$, to leave a single-peaked final state. The time–evolution of Eq. (1) is shown in **a**–**c**. The initial conditions (blue curves) and final states (black) are given in **d**–**f**. In all panels, $D=1$, $\gamma =10$, $p=1$, $L=1$, and *K* is a top-hat kernel (Eq. 19) with $\delta =0.1$ (so $\sigma =0.1/\sqrt{3}$). The labels ‘Start’ and ‘End’ in **d**–**f** refer respectively to the initial and final distributions of the corresponding space–time plots in **a**–**c** (Color figure online)

Numerics reveal that the system does indeed tend towards a single-peaked solution, where the width of the peak is approximately $\sqrt{2}\pi \sigma$ and the solution is zero elsewhere (Fig. 2). However, the asymptotic distribution is more flat-topped than the initial condition, owing to the fact that the initial condition arises from a moment closure approximation of $K*u$. This approximation reduces the analytic solution to a single Fourier mode, whereas the numerical solution could have arbitrarily many Fourier modes.

**Fig. 3:**
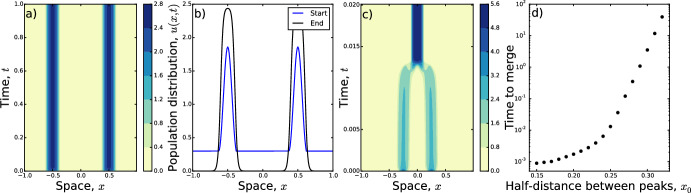
Similar to the single peak case (Fig. 2), when we start with two peaks of equal heights, surrounded by an area of constant density $\epsilon$, that area becomes sucked-up into the peak. We see this for $x_0=0.5$ and $\epsilon =0.2$ in **a**, **b**, where both peaks remain. For $x_0<0.5$, peaks merge, shown in **c** for $x_0=0.25$. The time to merge as a function of $x_0$ is given in **d**. Parameters *D*, $\gamma$, *p*, *L*, and *K* are as in Fig. 2

**Fig. 4:**
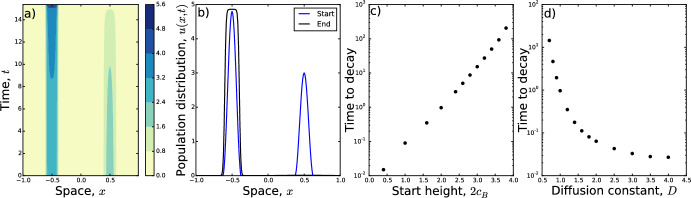
**a** Numerical solution of Eq. (5) with initial condition given by Eq. (23) with $c_B=1.5$. **b** Snapshots of the initial and final distributions from **a**. Notice that the smaller peak has decayed almost completely by $t \approx 15$. The graph in **c** is constructed from numerical solutions of Eq. (5) with initial condition given by Eq. (23) but with $c_B$ taking a variety of values, giving different start heights for the smaller peak (note that the start height is $2c_B$). Both **c** and **d** plot the time it takes for the smaller peak to decay to a maximum height of < 0.1. This increases exponentially as a function of the start height, explaining the appearance of long-transient multi-peaked solutions to Eq. (5) (**c**). Conversely, the decay time decreases as *D* is increased, showing how diffusion can speed up decay of the smaller peak (**d**). In **a**–**c**, $D=1$. In **d**, $c_B=1$. In all panels, $\gamma =10$, $p=1$, $L=1$, and *K* is a top-hat kernel (Eq. 19) with $\delta =0.1$. The value of $c_A$ is determined by Eq. (24)

**Fig. 5:**
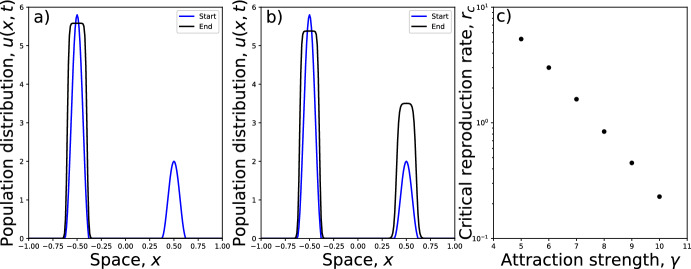
Effect of growth parameter. Examples are given in **a**, **b**, showing the initial condition (blue) and solution at time $t=10$ (black) where the parameters are $\gamma =10$, $D=1$, and $R=5$. In **a**, $r=0.23$ whereas **b** has $r=0.24$. This demonstrates a transition in long-term patterns, whereby the smaller peak decays for $r\le 0.23$ but grows for $r\ge 0.24$. In **c**, we observe that the transition point, $r_c$, decreases exponentially as the strength of attraction, $\gamma$, increases (Color figure online)

For our numerics, we use a forward difference approximation with $\Delta t = 10^{-5}$ and $\Delta x=0.01$. Each of the ‘End’ distributions in Figs. 2, 3, 4 and 5 have the property that $|u(x,T-1000\Delta t)-u(x,T)|<10^{-6}$, where *T* is time at which the ‘End’ distributions are calculated. The Python code we used to perform numerics is available at https://github.com/jonathan-potts/PottsPainter.

Finally note that, in the case $\epsilon =0.3$ (Fig. 2c, f), a second peak emerges around $x=\pm 1$ (which are identified due to the periodic boundaries, recalling that $L=1$). However, this decays by about $t=4$. We will return to this phenomenon of decaying secondary peaks in the next section.

## Twin Peaks

In this section, we examine situations where there are two peaks (results from which can be extended to multiple peaks). First, we look at situations where the peaks are the same height, then at cases where one peak is smaller than the other.

### Peaks of Identical Height

Similar to the single-peak case, here we want to understand whether it is energetically favourable for a solution to have no mass outside the two peaks. More precisely, we examine the energy of the following solution to Eq. (5), which is a critical point of *E*[*u*]

20 $$\begin{aligned} u_*(x)={\left\{ \begin{array}{ll} \epsilon +c_{\epsilon }\left[ 1+\cos \left( \frac{(x+x_0)\sqrt{2}}{\sigma }\right) \right] , &{} \text{ if }\,x \in \left( -x_0-\frac{\pi \sigma }{\sqrt{2}},-x_0+\frac{\pi \sigma }{\sqrt{2}}\right) , \\ \epsilon +c_{\epsilon }\left[ 1+\cos \left( \frac{(x-x_0)\sqrt{2}}{\sigma }\right) \right] , &{} \text{ if }\,x \in \left( x_0-\frac{\pi \sigma }{\sqrt{2}},x_0+\frac{\pi \sigma }{\sqrt{2}}\right) , \\ \epsilon , &{} \text{ otherwise. } \end{array}\right. } \end{aligned}$$

Here, $x_0 \in \left( \frac{\pi \sigma }{\sqrt{2}},\frac{L}{2}\right)$ is half the (shortest) distance between the centres of the two peaks. As in the single-peak case, we can use Eq. (7) to calculate

21 $$\begin{aligned} c_{\epsilon }=\frac{p-2L\epsilon }{2\sqrt{2}\pi \sigma }. \end{aligned}$$

A direct calculation using the definition of *E*[*u*] from Eq. (8) leads to

22 $$\begin{aligned} E[u_*]=\frac{\sqrt{2}L}{\pi \sigma }(\sqrt{2}\pi \sigma -L)\epsilon ^2 + \frac{\sqrt{2}p}{\pi \sigma }(L-\sqrt{2}\pi \sigma )\epsilon - \frac{p^2}{2\sqrt{2}\pi \sigma }. \end{aligned}$$

Since $\sqrt{2}\pi \sigma <L$, this is a negative quadratic in $\epsilon$. The unique turning point is a maximum at $\epsilon =\frac{p}{2L}$, so $E[u_*]$ is an increasing function of $\epsilon$ on the interval $[0,\frac{p}{2L}]$. Hence the minimum energy in the two-peaked case is where $\epsilon =0$, as with the one-peaked case. However, comparing the $\epsilon =0$ situation with one peak (Eq. 18), against that with two peaks (Eq. 22), we see that the single peak is a lower-energy solution. This suggests that we might also see a merging of the two peaks, as well as the mass outside the peaks tending to zero.

Indeed, in our numerical experiments, we saw a merging of peaks except in the special case where $x_0=0.5$, so that the initial peaks are evenly-spaced. Figure 3a, b shows an example where $x_0=0.5$ but $\epsilon >0$. Here two peaks remain but the mass outside those two peaks is absorbed into the peaks over time. Figure 3c gives an example of peak merging for $x_0<0.5$ whilst Fig. 3d shows how the time it takes for peaks to merge increases dramatically as $x_0$ increases towards $x_0=0.5$. Here, the time to merge is defined as the time at which the centre of the two initial peaks drops below 0.1. Whilst this is a rather arbitrary definition, other definitions lead to similar trends.

Notice that our energy analysis does not give direct insight into why merging does not happen for $x_0=0.5$. However, physical intuition suggests evenly-spaced peaks means that there is no ‘preferred’ direction for them to move in order to coalesce. Therefore they remain as two peaks. This symmetry of multi-peaked solutions was also shown in a similar model by Buttenschön and Hillen (2020), using group-theoretic arguments.

### Peaks of Differing Heights

In Sect. 4.1, we examined situations where there are two peaks with precisely equal height, finding that both peaks persisted indefinitely when they are evenly-spaced. However, we have already seen in Fig. 1 that when peaks are of different heights, the smaller one can shrink over time, whereas the larger one grows. If this continues indefinitely, the smaller peak could decay completely and only one peak would remain, although it might take a long time for this to happen.

Here, we seek to explain this phenomenon using our energy approach, ascertaining whether we should always expect a smaller peak to end up decaying to zero, or whether there are situations where two peaks remain. To this end, we examine steady state solutions with the following functional form

23 $$\begin{aligned} u_*(x)={\left\{ \begin{array}{ll} c_{A}\left[ 1+\cos \left( \frac{(x+x_0)\sqrt{2}}{\sigma }\right) \right] , &{} \text{ if }\,x \in \left( -x_0-\frac{\pi \sigma }{\sqrt{2}},-x_0+\frac{\pi \sigma }{\sqrt{2}}\right) \\ c_{B}\left[ 1+\cos \left( \frac{(x-x_0)\sqrt{2}}{\sigma }\right) \right] , &{} \text{ if }\,x \in \left( x_0-\frac{\pi \sigma }{\sqrt{2}},x_0+\frac{\pi \sigma }{\sqrt{2}}\right) \\ 0, &{} \text{ otherwise. } \end{array}\right. } \end{aligned}$$

In this case, we can use Eq. (7) to calculate

24 $$\begin{aligned} c_{A}=\frac{p-2L\epsilon }{\sqrt{2}\pi \sigma }-c_B. \end{aligned}$$

We see immediately that, in order for $c_A$ and $c_B$ to be non-negative, we must have $c_A,c_B \in \left[ 0,\frac{p}{\sqrt{2}\pi \sigma }\right]$. A direct calculation using the definition of *E*[*u*] from Eq. (8) leads to

25 $$\begin{aligned} E[u_*]=-2\sqrt{2}\pi \sigma c_B^2+2pc_B-\frac{p^2}{\pi \sigma \sqrt{2}}. \end{aligned}$$

This is a negative quadratic in $c_B$ with critical point at $c_B=c_A=\frac{p}{2\sqrt{2}\pi \sigma }$. Therefore the energy minima occur either when $c_B=0$, $c_A=\frac{p}{\sqrt{2}\pi \sigma }$ or $c_A=0$, $c_B=\frac{p}{\sqrt{2}\pi \sigma }$. In other words, they occur when there is just one peak. Consequently, away from the critical point where $c_A=c_B$, we would expect the smaller peak to slowly decay to zero over time, leaving just one peak. Indeed, this is what we see in numerical solutions of Eq. (5) (e.g. Fig. 4a,b). However, the time it takes for the smaller peak to decay can be very large (Fig. 4c). This is exacerbated by decreasing the diffusion constant, *D* (Fig. 4d). Here, numerics hint that, as $D \rightarrow 0$, the decay time may tend to infinity, meaning that the second peak may persist indefinitely if there is no diffusion to allow the smaller to seep into the larger.

### Including Population Growth

So far, we have studied a system where the population size remains constant. This assumes that there are negligible births or deaths on the timescales that we are studying. Our focus has been on examining the difference between long transients and asymptotic solutions. However, in any real biological system, the effect of births and deaths will become non-negligible at some point in time. Therefore there is a limit to which transient solutions in these systems are biologically realistic: if the transients persist for too long, it will become necessary to account for the effect of births and deaths in any biologically meaningful model.

We therefore examine the extent to which incorporating growth might enable a second peak to persist, by solving the following equation numerically

26 $$\begin{aligned} \frac{\partial u}{\partial t}=D\frac{\partial ^2 u}{\partial x^2}-\gamma \frac{\partial }{\partial x}\left[ u\frac{\partial }{\partial x}(K*u)\right] +ru\left( 1-\frac{u}{R}\right) , \end{aligned}$$

with initial conditions given by Eq. (23).

Depending upon the values of $\gamma$, *D*, *R*, and $c_B$, we found that there is a critical value $r=r_c$ above which the second hump persists, and below which it decays. Figure 5a, b shows this in the case $\gamma =10$, $D=1$, $R=5$, $c_B=1$, whereby $r_c\approx 0.23$. Figure 5c demonstrates how $r_c$ depends upon the aggregation strength $\gamma$: the greater the aggregation strength, the higher the required growth rate to enable a second peak to persist.

## Discussion

Distinguishing between asymptotic solutions and long transients in numerical PDEs is a thorny issue, with perhaps no one-size-fits-all solution. Typically, researchers decide that a solution has reached an asymptotically-stable state when some measure (e.g. the change in $L^p$ norm for some $p \in [1,\infty ]$) is below a small threshold value (see e.g. Burger et al. 2014; Giunta et al. 2022a; Schlichting and Seis 2022). However, this means that if transient solutions are changing slower than this threshold value then they will be mistaken for asymptotically-stable solutions. Therefore it is valuable to have some analytic insight to guide the user as to whether the solution is (or is likely to be) a long transient or an asymptotically-stable solution.

Here, we have provided such a deductive technique for the aggregation–diffusion equation in Eq. (1). Rather than studying this equation directly, we instead study an approximation given in Eq. (5). This approximate formulation is simple enough to solve for steady state solutions. It also possesses an energy functional, which allows us to search for local minimum energy solutions amongst the steady state solutions, an approach employed successfully in a previous multi-species study (Giunta et al. 2022b). Our hypotheses are first that these local minimum energy solutions are stable solutions to Eq. (5), whereas other steady states are not; and second that this categorisation carries over to the steady states of Eq. (1). In the examples we tested, numerical experiments confirmed these hypotheses, with the sole exception of twin-peaked solutions where the peaks are of identical height and evenly-spaced. We therefore conclude that this method is a useful way for guiding users (i.e. those wanting to solving Eq. 1 numerically) as to whether a solution they are observing is likely to be stable or not, whilst also recommending that they verify these calculations up with numerical experiments.

Regarding the examples we tested, we found two main results: first, that stable aggregations are likely to resemble compactly-supported solutions, rather than being non-zero everywhere; second, that multi-peaked solutions will always be transient unless either $D=0$ or the peaks are precisely the same height and evenly-spaced. In addition to these central messages, further numerical investigations revealed that these twin-peaked transient solutions can be arbitrarily long-lived if the peaks are arbitrarily close to being evenly-spaced (Fig. 3) and the heights of these peaks are arbitrarily similar (Fig. 4).

That said, the consideration of very long transients in a model that operates on timescales where births and deaths are negligible is not terribly realistic, so we also examined the effect of adding a small amount of (logistic) growth. We found that arbitrarily small amounts of growth will not stop the smaller peak from decaying. However, there appears to be a critical growth rate, dependent upon the model parameters, below which the smaller peak will decay and above which it will grow (Fig. 5). Therefore, if long transients appear when using Eq. (1) to model biological aggregation, it is valuable to think about the effect of net reproductive rate in the system being modelled, and whether this is sufficient to arrest the decay of the smaller peak.

Whilst our principal equation of interest is Eq. (1), it is worth noting that our approximate analytic techniques can also be applied to various other equations. For example, the cell adhesion equations introduced in Armstrong et al. (2006) have a very similar functional form that can usually be formally related to Eq. (1) or modifications thereof (Painter et al. 2023). Chemotaxis equations are also somewhat similar to Eq. (1), but here the non-local self-interaction is replaced with a diffusing chemical. The organisms interact with the chemical rather than directly with one another. It turns out that the resulting models are equivalent to a type of aggregation-diffusion equation with advection that is nonlocal in both space and time (Knútsdóttir et al. 2014; Shi et al. 2021). This contrasts with Eq. (1), which is nonlocal in space alone. However, similar patterns are observed in these systems, including long-transient multi-peaked solutions similar to those studied here (Potapov and Hillen 2005). We also note that the moment closure we use in Eq. (1) leads to a fourth-order PDE of Cahn–Hilliard type (Novick-Cohen 2008), for which there is a long history of studies on metastability (Bates and Xun 1994; Reyna and Ward 1995; Scholtes and Westdickenberg 2018) and has recently been proposed in the context of biological aggregations (Falcó et al. 2023).

Passing from the non-local aggregation–diffusion equation (1) to the fourth-order local equation (5) involved two simplifying assumptions: the limit $D/\gamma \rightarrow 0$, and the moment closure approximation (3). However, these assumptions were concurrently applied, in that no definitive order was invoked. Separated out, two distinct intermediate models emerge: a non-local hyperbolic equation, if we first apply the limit $D/\gamma \rightarrow 0$, or a fourth-order local parabolic equation (with nonlinear diffusion) if we first assume (3). Deeper insights may be gained into the connection between models by investigating these distinct passages in detail. In particular, it would be of interest to explore the extent to which the two forms for the intermediate model exhibit qualitatively distinct properties from either the nonlocal aggregation–diffusion equation (1), or its approximation (5).

Following on from these observations, it is worth noting that while the version of the aggregation–diffusion equation that we study involves linear diffusion, the combined assumptions ‘convert’ this into a nonlinear quadratic diffusion of the form $(uu_x)_x$. In fact, there has also been considerable interest in nonlocal aggregation-diffusion equation that include nonlinear diffusion from the outset, i.e. $u_{xx}$ replaced with $(uu_x)_x$ in Eq. (1). An advantage of this formulation is that Eq. (1) has the form $u_t=[u(D-\gamma K*u)_x]_x$, making it amenable to a analysis without taking the limit $D/\gamma \rightarrow 0$. This fact has been exploited, for example, by Ellefsen and Rodriguez (2021) and Carrillo et al. (2018). However, here we have chosen to focus on linear diffusion is important to study as it often arises naturally from models of organism movement (Armstrong et al. 2006; Potts and Schlägel 2020; Painter et al. 2023). Future work on the nonlinear case could reveal analytic insights about the effect of *D* vs. $\gamma$ on asymptotic patterns, which we were only able to examine numerically in this study. Likewise, the consideration of higher spatial dimensions (particularly 2D and 3D) would be a biologically-important topic for future study, but we caution that the pattern formation properties can be rather more complicated (Jewell et al. 2023).

## Data Availability

This study has no associated data. Code for numerics is available at https://github.com/jonathan-potts/PottsPainter.
